# Serum Uric Acid in Roma and Non-Roma—Its Correlation with Metabolic Syndrome and Other Variables

**DOI:** 10.3390/ijerph15071412

**Published:** 2018-07-04

**Authors:** Jana Petrikova, Martin Janicko, Jan Fedacko, Sylvia Drazilova, Andrea Madarasova Geckova, Maria Marekova, Daniel Pella, Peter Jarcuska, HepaMeta Team

**Affiliations:** 1 1st Department of Internal Medicine, Faculty of Medicine, PJ Safarik University, 040 11 Kosice, Slovakia; jana.petrikova@upjs.sk (J.P.); martin.janicko@gmail.com (M.J.); daniel.pella@upjs.sk (D.P.); 2 Louis Pasteur University Hospital, 040 01 Kosice, Slovakia; 3 Department of Gerontology and Geriatrics, Faculty of Medicine, PJ Safarik University, 040 01 Kosice, Slovakia; jan.fedacko@upjs.sk; 4 Air Force Military Hospital, 040 86 Kosice, Slovakia; 5 Department of Internal Medicine, Poprad Hospital, 058 01 Poprad, Slovakia; drazilova.s@nemocnicapp.sk; 6 Department of Health Psychology, Faculty of Medicine, PJ Safarik University, 040 11 Kosice, Slovakia; andrea.geckova@upjs.sk; 7 Department of Medical and Clinical Biochemistry, Faculty of Medicine, PJ Safarik University, 040 11 Kosice, Slovakia; maria.marekova@upjs.sk

**Keywords:** Roma population, metabolic syndrome, uric acid

## Abstract

*Background*: The Roma population is one of the major marginalized groups in Europe, having higher incidence of all spectrums of disease and a shorter life expectancy. Yet, the reasons for higher morbidity and its exact prevalence were not properly studied. *Objectives*: The objective of our study was to compare the frequency of metabolic syndrome (MetS) in Roma people to the non-Roma population in Slovakia, and to compare levels of uric acid and its correlation with components of metabolic syndrome. *Methods*: A group of 452 Roma people aged 18–55 years, was compared to a control group of 403 non-Roma people. The data were obtained by questionnaire, anthropometric measures, and analyzed blood and urine samples *Results*: The prevalence of MetS was significantly higher among Roma participants (131; 29.6%) compared with non-Roma participants (80; 20.1%), *p* = 0.001. Roma people significantly more often fulfilled obesity and low high-density lipoprotein (HDL) criteria of MetS (257, 58.9% vs. 180, 45.8%, *p* < 0.0001, and 312, 70.0% vs. 140, 34.9%, *p* < 0.0001). There was no difference in the triacylglycerols (TG), glycemia or blood pressure (BP) criteria of MetS. The Roma also presented with greater levels of high sensitivity C-reactive protein (hs-CRP). Baseline levels of uric acid (UA) among the Roma population were significantly lower compared with the majority population (226.54 ± 79.8 vs. 259.11 ± 84.53) (*p* < 0.001). The levels of UA significantly correlated with fulfilled criteria of MetS. Univariate regression showed that UA is a significant predictor of MetS in the whole cohort (unadjusted odds ratio (OR) 1.005; 95% CI 1.004–1.007; *p* < 0.0001) also after the adjustment for age, sex, and ethnicity (adjusted OR 1.008; 95% CI 1.005–1.010; *p* < 0.0001). *Conclusions*: We were able to show that prevalence of MetS among the Roma is higher than in the majority population. Moreover, the uric acid levels are significantly lower in the Roma group as well as when it comes to a cohort with MetS. Levels of UA, besides others, depend on ethnicity, age, and sex.

## 1. Introduction

Because the Roma population is one of the major ethnic minority populations in Europe, its health-related issues should be studied carefully. Mainly because past studies suggest that this marginalized group suffers from an increased prevalence of contagious and non-contagious illnesses in the community, and importantly, a shorter life expectancy than the national averages. The intentions of authorities across Europe to solve these health inequalities have been relatively poor [1,2] and, in comparison to the majority population, the Roma have reduced access to health care as well as lower rates of education and employment in every country where they live [3].

Serum uric acid is a final enzymatic product of purine metabolism in humans. It is suggested that the increased levels of uric acid are linked with metabolic syndrome (MetS), and they may have a common pathophysiology [4]. In addition to MetS, higher concentrations of uric acid are studied as an important player in a pathophysiology of a range of cardiovascular conditions [5].

Obesity, dyslipidemia, hypertension, and insulin resistance are a group of risk factors that define metabolic syndrome [6]. When they appear together, the risk of developing cardiovascular disease (CVD) and diabetes is elevated [7]. Past results suggest that well defined components of MetS cannot explain all CVD cases observed in those patients. Consequently, many other risk components, such as inflammatory markers, microalbuminuria, hyperuricemia, and disorders of coagulation, have been debated regarding whether to be included in the MetS definition [8,9,10,11,12]. A novel study brings data on the role of Uric Acid (UA) in the development of renal alteration [13]. The incidence of MetS are increasing worldwide. Metabolic syndrome patients have at least a 2-fold increased risk for CVD. Moreover, when it comes to type 2 diabetes, the risk in both genders is increased by about 5-fold [14], and when speaking about coronary heart disease (CHD), the risk is 2–3 times higher [15]. Therefore, a major challenge for health care professionals facing an epidemic of people who are overweight and have a sedentary lifestyle, should be the early identification, treatment, and prevention of metabolic syndrome [16]. Ethnic minorities are known to have more increased medical risks than the majority populations [1,17,18]. In the Slovak Republic, the Roma are the second-largest minority group [19]. According to professional demographic estimates, roughly 430,000 Roma live in Slovakia, which represents about 8% of the total population of the country [3]. The risk of atherogenesis in the Roma minority has noticeably grown as some results suggest. This might be as a result of adverse factors, such as a rise in the prevalence of obesity, hypertension, smoking, and deficiency in protective substances, leading to dyslipidemia, hyperinsulinemia, cardiovascular diseases, metabolic syndrome, and diabetes [20]. Because previous studies have shown a higher mortality and a shorter life span for the Roma than for the non-Roma [3], a high prevalence of metabolic syndrome among groups of lower socioeconomic status should demand for an urgent and efficient public health responses [2].

The aim of this study was to determine the prevalence of metabolic syndrome in the Roma population compared with the non-Roma population in the eastern part of Slovakia, and to determine the levels of uric acid and its correlation to metabolic syndrome components and other variables in the same population sample.

## 2. Materials and Methods

Data from the cross-sectional population-based HepaMeta study conducted in Slovakia in 2011 were used. The aim of the project was to explore the health, health related behavior, social and economic parameters, and health care accessibility in Roma people in segregated settlements and to compare it to the non-Roma population. All subjects gave their informed consent for inclusion before they participated in the study. The study was conducted in accordance with the Declaration of Helsinki, and the protocol was approved by the Ethics Committee of the Faculty of Medicine at Šafárik University in Košice (no. 104/2011). Participation in the study was fully voluntary and anonymous.

Out of 710 selected non-Roma people, 403 chose to participate (response rate 56.8%). The sample consisted of 452 Roma people (mean age = 34.7; 35.2% men) aged 18–55 and 403 non-Roma people (mean age = 33.5; 45.9% men) aged 18–55. The Roma in selected settlements were recruited by local Roma community workers. Respondents from the majority population were randomly selected from a list of patients from general practitioners (GP’s). The data were collected via questionnaire, anthropometric measures, and blood sample analyses after an overnight fasting. The samples were collected and modified (centrifuged, frozen) at a biochemical laboratory of the Department of Medical and Clinical Biochemistry and LABMED, Faculty of Medicine, P. J. Šafárik University, Košice.

### 2.1. Measures

Clinical biochemistry tests for determination substrates were as follows: cystatin C (cysC), creatinine, minerals (serum iron level [Fe]), proteins (ferritin and high sensitivity C-reactive protein [hs-CRP] as a risk factor), enzymes (ALT—alanine-aminotransferase; AST—aspartate-aminotransferase; GGT—gamma-glutamyl transferase), glucose, uric acid (UA), and lipid parameters (TAG or TG—triacylglycerols; TC—total cholesterol; HDL-C—high-density lipoprotein cholesterol; low-density lipoprotein cholesterol—LDL-C; apoA—apolipoprotein A; apoB100—apolipoprotein B100). All of the biochemical parameters were determined by routine biochemical methods on analyzer ADVIA 2400 or 1650. Ferritin was measured by chemiluminescent immunoassay (CLIA) on analyzer ADVIA Centaur (Siemens, Erlangen, Germany). The methodology is described in detail elsewhere [21].

The International Diabetes Federation standard criteria were used for the determination of metabolic syndrome (MetS) [15]. The patients were considered to have MetS when the central obesity (waist circumference ≥94 cm for males and ≥80 cm for females, or body mass index [BMI] >30 kg/m^2^) plus any two of the four following factors were present: raised TG ≥1.7 mmol/L (150 mg/dL) or specific treatment for this lipid abnormality; reduced HDL-C <1.03 mmol/L (40 mg/dL) in males and <1.29 mmol/L (50 mg/dL) in females, or specific treatment for these lipid abnormalities; raised systolic blood pressure ≥130 mmHg or diastolic blood pressure ≥85 mmHg, or treatment of previously diagnosed hypertension; raised fasting plasma glucose ≥5.6 mmol/L (100 mg/dL), or previously diagnosed type 2 diabetes. Blood pressure (BP) was taken using the Omron M3 digital automatic blood pressure monitor after 5 min of rest in a sitting position. The mean value of three blood pressure measurements was used in the analysis. The limit for the normal systolic blood pressure was <130 mmHg or normal diastolic blood pressure <85 mmHg.

The medical history data were taken from the participants’ records in the GP’s office. The social and economic data were obtained through questionnaires and were self-reported by the participants. The Roma participants answered the questionnaires with the help of research assistants, and the non-Roma people answered the questionnaires individually, but help was provided upon request. The obtained information were as follows: employment (binary), any current smoking (binary), more than six standard drinks of alcohol per month (binary), imprisonment (binary), physical activity more than once per week (binary), education classified into three categories (elementary, apprenticeship, and higher education), and poverty (binary), which was an aggregate variable that contained the inability to pay one item of the following: rent, loan payment, healthcare, and energies and other expenses.

### 2.2. Statistical Analysis

The categorical data are presented as absolute and relative counts. The interval data is presented as mean ± standard error of mean. Additionally, due to the easier visual comparison, non-parametric interval variables are presented as mean and standard errors of means (SEM), because of the sufficient number of participants. The statistical significance of differences between the categorical data was assessed by the chi-square test. The differences in the interval variables were tested by *t*-test in the case of two groups, and the ANOVA or Kruskall–Wallis test with post-hoc Tukey HSD in the case of a multiple group comparison, while respecting the tests’ presumptions. The only variables tested as nonparametric were AST, ALT, GGT, and hsCRP.

After baseline comparisons, we used the Pearson correlation to evaluate the correlation between UA and other biochemical parameters. The Pearson correlation was used also to evaluate the collinearity of the predictors in the multivariate regression, where we used the correlation coefficient cut-off of 0.7 to flag significant collinearity.

Lastly, we performed a multivariate logistic regression with MetS as the dependent variable, to explore the independent predictive value of the potentially significant variables from previous analyses.

Since this was an exploratory study, no prior power analysis was performed. The post hoc power analysis for the difference in MetS prevalence between the Roma and non-Roma revealed a power of 91.4%.

All of the statistical analyses were performed by SPSS statistical package (version 22, IBM Corp., Armonk, NY, USA).

## 3. Results

### 3.1. Description of the Study Population

In this observational study, we examined a total of 442 Roma participants and 399 non-Roma participants (missing data for 10 participants in the Roma and 4 participants from the non-Roma group to categorize the presence of MetS). Prevalence of MetS was significantly higher among the Roma participants (131; 29.6%) compared with the non-Roma (80; 20.1%), *p* = 0.001. The Roma people significantly more often fulfilled obesity and a low HDL criteria of MetS (257; 58.9% vs. 180, 45.8%; *p* < 0.0001 and 312; 70.0% vs. 140; 34.9%; *p* <0.0001). There was no difference in the TG, glycemia, or BP criteria of MetS. Table 1 summarizes the values of other variables in the study cohort by ethnicity.

The Roma population representatives were slightly but significantly older and included less men. Among the basic biochemical characteristics, the Roma people had lower levels of creatinine, UA, and hepatic enzymes activity (AST, ALT, and GMT). Also, the Fe levels were importantly lower in the Roma population. Despite having a significantly greater BMI, the Roma people statistically differed in lower levels of TCH, ApoA, and HDL. On the other hand, they presented with greater levels of hs-CRP. Surprisingly, there was no difference in the baseline glucose, TG, and LDL levels among both of the studied populations. They also did not differ in waist to hip ratio (WHR) and BP. Socioeconomic variables were significantly more prevalent among the Roma population—they reported a higher incidence of poverty, more smoking, and lower incidence of all levels of education. The occupational status was also lower among Roma. Physical activity and imprisonment was importantly more frequent among the Roma.

#### 3.1.1. Baseline Parameters of Study Cohorts

The differences among the four categories (Roma and non-Roma, with and without MetS) are summarized in Table 2.

The post hoc analysis shows significant differences between the Roma and non-Roma participants in both the MetS positive and negative groups. Glycemia was higher in the participants with MetS, regardless of ethnicity. Uric acid levels were significantly increased in both the Roma and non-Roma participants with MetS, but were overall higher in the non-Roma group. AST activity was higher in the non-Roma group overall, but no difference in the MetS status was observed. ALT activity was higher in MetS group, only in the non-Roma. GMT activity was higher in the MetS group both in the Roma and non-Roma participants. Creatinine was increased in the MetS group, only in the non-Roma participants. The serum level of albumin was decreased only in the non-Roma with MetS, and no difference was observed in the Roma. Both creatinine and cystatin C were higher in the MetS group only in the non-Roma, and no difference was observed among the Roma participants. As expected, all of the lipid parameters were significantly different between the MetS positive and negative patients in both the Roma and non-Roma. High sensitivity CRP levels were significantly increased in the MetS group, both in the Roma and non-Roma, but in the Roma with MetS they were even higher than the non-Roma with MetS. The serum iron levels were decreased, and the ferritin levels were increased in the MetS group both in the Roma and non-Roma.

The Roma participants with MetS were older than the non-Roma counterparts, but there was no age difference between the Roma and non-Roma without MetS. Also, no difference in gender was observed between the MetS positive and negative groups. As expected, people with MetS had a higher BMI, and the Roma with MetS had an even higher BMI compared with the non-Roma with MetS. There was no difference in the BMI between the Roma and non-Roma without MetS. However, no difference was observed in the WHR whatsoever. Both the systolic and diastolic blood pressure were increased in all of the people with MetS, but the diastolic blood pressure was also higher in the non-Roma without MetS, compared with their Roma counterparts.

There was no difference in employment, alcohol consumption, smoking, or physical activity between the MetS positive and negative Roma, as well as the non-Roma. However, both the Roma and non-Roma who had MetS were more often impoverished. Furthermore, the non-Roma who were less educated had a higher prevalence of MetS, but this was not observed in the Roma participants.

#### 3.1.2. Uric Acid and Ethnicity

The baseline levels of UA among the Roma population were significantly lower compared with the majority population (226.54 ± 79.8 vs. 259.11 ± 84.53) (*p* < 0.0001). The Roma ethnicity increased the UA level in multivariate linear regression by 0.163; the female sex decreases the level of UA by 0.438 and age increases level of UA by 0.092. Levels of UA, besides others, depend on ethnicity, age, and sex, which are independent from each other and all together predict 23.5% of the UA variability.

### 3.2. Uric Acid Levels and Its Relationship to Demographic and Socioeconomic Variables

We observed a significant difference in the UA between the sexes in both the Roma and non-Roma participants (Roma males 265 ± 7.1 vs. females 205 ± 3.8 μmol/L, *p* < 0.0001; non-Roma males 304 ± 6.2 vs. females 220 ± 4.2 μmol/L, *p* < 0.0001). There was also a weak direct correlation between the UA and age in the Roma but not non-Roma.

We explored several socio-economic variables regarding the UA levels. We have discovered that UA levels were almost significantly higher in the impoverished Roma, but not the non-Roma. They were significantly increased in the non-Roma and almost significantly in the Roma regular alcohol consumers, and lastly in the Roma with a history of incarceration. No difference in the UA levels between the previously imprisoned and not imprisoned participants was observed when grouped by sex, probably due to the low numbers of participants in the subgroups. No difference in other variables, particularly physical activity or smoking, was discovered (Table 3).

### 3.3. Uric Acid and Its Relationship to Biochemical Variables

Using a simple correlation model of continuous variables, we were able to show that the levels of UA correlate with other biochemical parameters, as shown in Table 4. Almost all of the correlations were very significantly positive in both the Roma and non-Roma, except for ALT in the Roma and Fe in the non-Roma. The correlations between UA and HDL, and UA and apolipoprotein A were inverse, all other correlations were direct.

### 3.4. Uric Acid and Its Relationship to Metabolic Syndrome

**Figure 1:**
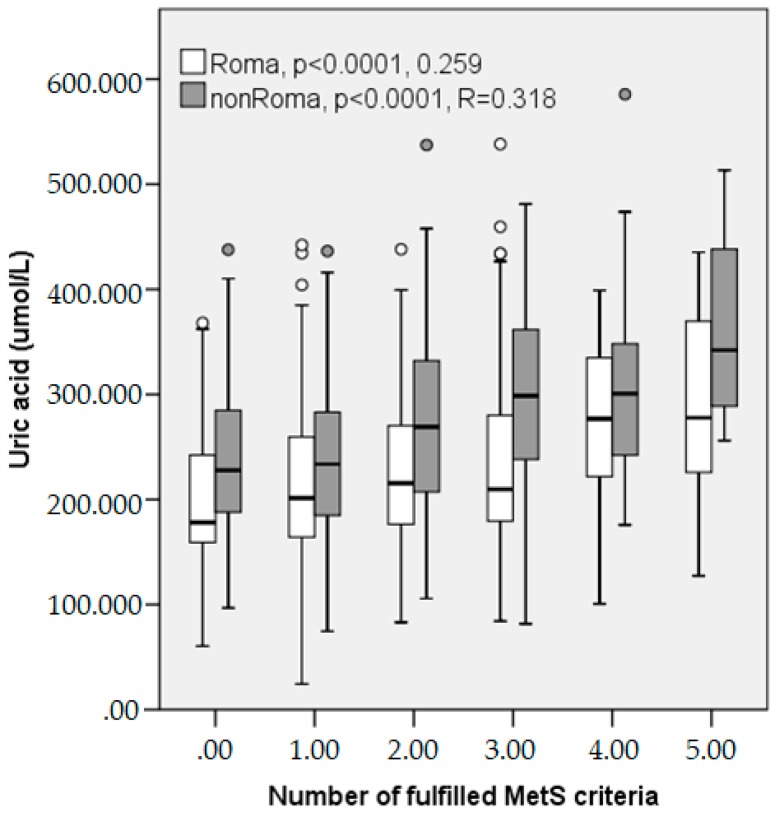
Uric acid levels in Roma and non-Roma people by number of fullfiled MetS criteria. *p* for trend.

As already shown in Table 2, the participants with MetS, both Roma and non-Roma, had higher levels of UA. We observed this difference in each individual MetS criterium, except for HDL (Table 5). There was also a significant trend toward the increase of uric acid levels with the number of fulfilled MetS criteria (Figure 1).

Univariate regression showed that UA is a significant predictor of MetS in the whole cohort (unadjusted OR 1.005; 95% CI 1.004–1.007; *p* < 0.0001) also after the adjustment for age, sex, and ethnicity (adjusted OR 1.008; 95%CI 1.005–1.010; *p* < 0.0001). The fully adjusted multivariate model (Table 6) included all variables where we found significant differences between the participants with and without MetS, in both the Roma and non-Roma. We excluded the variables used as a definition of MetS (BP, TG, HDL, glycemia, and BMI). We also detected a high collinearity between the total cholesterol, LDL, and Apolipoprotein B100, therefore, we included only the total cholesterol. Uric acid remains a significant and independent predictor of MetS in the whole cohort. The odds of MetS were also increased by age, male sex, elevation of GMT, hsCRP, total cholesterol, and the decrease of ApoA. Poverty was almost a significant predictor of MetS in this model. Interestingly, Roma ethnicity was not a significant predictor of MetS in this model, along with serum iron and ferritin.

## 4. Discussion

Research initiatives during the past years have focused on contagious diseases, genetic topics, and child health issues in the Roma population as emerging topics. Nowadays, trends have shifted to non-communicable diseases, chronic illnesses, and associated risk factors that would give an answer to the poorer health outcomes of the Roma population.

The results of our study suggest that there is a higher prevalence of metabolic syndrome in the Roma population. Interestingly, the Roma in our study had significantly lower levels of UA when comparing the baseline and subpopulations with MetS.

The Roma population representatives of our study cohort were slightly but significantly older and included less men. Among the basic biochemical characteristics, the Roma people had lower levels of creatinine and hepatic enzymes activity (AST, ALT, and GMT). Also, the Fe levels were importantly lower in the Roma population. Despite having a significantly greater BMI, the Roma people failed to reach significant differences in WHR and BP. Moreover, they had lower levels of TCH, ApoA, and HDL, all pointing towards cardiovascular risk factors. Surprisingly, there was no difference in the baseline glucose, TG, and LDL levels among both of the studied populations. On the contrary, the Roma presented with greater levels of hs-CRP. The baseline levels of UA were lower in the Roma representatives. Such a phenomenon may be a result of a genetic background showing a higher frequency of *SLC22A12* variants causing renal hypouricemia 1 in the Czech and Slovak Roma population [22]. The Roma cohort differed in socioeconomic variables—they self-reported a higher incidence of poverty, more smoking, and lower incidence of all levels of education. Consequently, the Roma people were less employed. All these variables have been previously reported by others [23]. Physical activity and imprisonment was more frequent among the Roma.

Within the population described in this study, the prevalence of MetS was significantly higher among the Roma participants compared with the non-Roma. In the Hungarian environment, a more than 50% incidence of MetS in the Roma population was reported [24]. This result might rather be an overestimation. Another Hungarian study showed the incidence of MetS up until 38%, with no difference between the Roma and the major Hungarian population [25]. Previous Slovak data reported a 20% incidence of MetS, with a significant difference between the Roma and the majority population in Slovakia [26]. This may indicate a rising pattern in MetS incidence among the Roma over a decade. Further analysis showed significant differences between the Roma and non-Roma participants in both the MetS positive and negative groups. It is well accepted that the prevalence of MetS rises with age. Our subjects with MetS were significantly older (*p* < 0.0001). No difference in gender was observed between the MetS positive and negative groups. Glycemia as one of the MetS determinants was higher in participants with MetS (5.4 and 5.16 vs. 4.61 and 4.74), regardless of ethnicity. Uric acid levels were significantly increased in both the Roma and non-Roma participants with MetS (251.61 vs. 303.64) but were overall higher in the non-Roma group. The GMT activity was higher in the MetS group both in the Roma and non-Roma participants. This is probably attributed to non-alcoholic fatty liver disease, as NAFLD is the hepatic manifestation of metabolic syndrome. The serum level of albumin was decreased only in the non-Roma with MetS, and no difference was observed in the Roma. Another potential novel biomarker associated with MetS and obesity are cystatinC and creatinine levels. Both were studied by a group, Ying X et al., who revealed that the CysC was more closely associated with the presence of MetS, as compared with serum creatinine or eGFRCKD-EPI. CysC was positively correlated with BMI, and more strongly and positively correlated with waist circumference and inflammation in their study [27]. Our data showed that both creatinine and cystatin C were significantly higher in the MetS group only in the non-Roma, and no difference was observed among the Roma participants. Lipid profile analyses in our study population significantly differed between the MetS positive and negative individuals in both the Roma and non-Roma. The HDL cholesterol levels are statistically lower, which was previously also reported by others in the Roma population. The LDL cholesterol and total cholesterol levels were significantly lower in the Roma subjects with metabolic syndrome, which is in contrast with previous reports [26]. HDL is an established risk factor for coronary artery disease, and recent studies have shown that for predicting a risk of coronary heart disease, ApoB1 and ApoA might be preferable laboratory parameters to the traditional lipid measures [28]. In our study, the Roma with MetS have significantly decreased levels of ApoB1 and decreased levels of ApoA, which may be a possible explanation for the increased CVD morbidity and mortality among the Roma in comparison with the non-Roma. Preventive cardiology recently focuses on inflammation as a popular theory, and among studied inflammatory markers, the acute-phase response marker C-reactive protein (measured by high sensitive technique—hsCRP) has been shown to anticipate the risk of future myocardial infarction beyond the contribution of traditional risk factors [29]. High sensitivity CRP levels were significantly increased in the MetS group both in the Roma and non-Roma, but in the Roma with MetS they were even higher than the non-Roma. MetS is increasingly being viewed as an inflammatory disease. It is well-defined that the serum iron level has a positive correlation with the risk of obesity. On the other hand, the connection between the increased serum ferritin levels, a marker of iron metabolism, which has recently emerged as a biomarker of a chronic low-grade inflammation, and the metabolic syndrome remains questionable. The serum iron levels were decreased, and the ferritin levels were increased in the MetS group both in the Roma and non-Roma, which is consistent with other studies [30,31]. As expected, the people with MetS had a higher BMI, the Roma with MetS had an even higher BMI compared with the non-Roma with MetS. There was no difference in the BMI between the Roma and non-Roma without MetS. However, no difference was observed in the WHR whatsoever. Both the systolic and diastolic blood pressure were increased in all of the people with MetS, but the diastolic blood pressure was also higher in the non-Roma without MetS, compared with their Roma counterparts. There was no difference in employment, alcohol consumption, smoking, or physical activity between the MetS positive and negative Roma, as well as non-Roma. However, both the Roma and non-Roma who had MetS were more often impoverished. Furthermore, the non-Roma who were less educated had a higher prevalence of MetS, but this was not observed in the Roma participants. One of the possible explanations might be that the level of poverty is not a determining factor in MetS development. On the other hand, there is a possibility that the development of MetS is mostly attributed to genetic factors. Some papers have shown that a predisposition for metabolic syndrome is linked to the composition of the mother’s milk during the first five days of life [32]. The predisposition of Romanies to acquire the metabolic syndrome may also be associated with a ‘thrifty gene’ hypothesis. The hypothesis suggests that, in a population that is undergoing evolution from malnutrition to better nutrition, they are very likely to be obese with impaired glucose and fat tolerance, as well as having heart disease [33].

Higher serum uric acid levels are a common laboratory result in patients with metabolic syndrome/obesity, hypertension, kidney disease, and cardiovascular events. Moreover, recent analysis suggests that the high-fructose diet in the United States, as one of the causes of hyperuricemia, may be contributing to the metabolic syndrome/obesity epidemic, diabetes, hypertension, kidney disease, and cardiovascular disorder [34,35]. Baseline levels of UA in the Roma population differed statistically, surprisingly being lower despite a higher incidence of MetS and its components in this cohort. One can speculate whether it is attributed to the genetic background or important diet differences.

Gender differences of UA are well accepted phenomenon. We observed significant difference in the UA between the sexes in both the Roma and non-Roma participants (Roma males 265 ± 7.1 vs. females 205 ± 3.8 μmol/L; *p* < 0.0001; non-Roma males 304 ± 6.2 vs. females 220 ± 4.2 μmol/L; *p* < 0.0001). There was also a weak direct correlation between UA and age in the Roma, but not the non-Roma. Serum uric acid levels in our study were importantly higher in the subjects with MetS and were significantly correlated with the fulfilled criteria of MetS, except from a HDL level. There was also a significant trend toward the increase of uric acid levels with the number of fulfilled MetS criteria. This finding is in agreement with some other results [36,37,38]. Several past studies suggest that the high level of plasma TG are related to hyperuricemia [39,40,41]. Hyperuricemia is often linked with arterial hypertension [42]. In a recent study, hyperuricemia was reported in 25–40% of the untreated hypertensive and 75% of the malignant hypertensive subjects [41]. Novel studies have suggested that the serum uric acid level is an independent risk factor for the development of hypertension [43]. Hyperuricemia is thought to be a result of hyperinsulinemia in metabolic syndrome and is attributed to decreased uric acid excretion in kidney dysfunction [44]. In various studies, it is not acknowledged as a main mediator of metabolic syndrome, renal disease, and cardiovascular disorder development. However, novel explorations with compelling evidence have changed this traditional view and support an independent link between hyperuricemia and an increased risk of metabolic syndrome, NAFLD, diabetes, hypertension, kidney disease, and cardiovascular disorders. Despite these new findings, there is still controversy regarding the precise role of uric acid in inducing these diseases [45].

After adjusting for confounding factors such as age and sex, a model was calculated explaining the 23.5% variability of UA. The levels of UA, besides others, depend on ethnicity, age, and sex, which are independent from each other and they were not significantly different in the groups regarding the socio-economic variables. Regular alcohol consumption resulted in higher levels of UA in the non-Roma participants, but the difference failed to reach statistical significance in the Roma subjects. We have discovered that the UA levels were almost significantly higher in the impoverished Roma, but not the non-Roma. The level of poverty seems not to influence the incidence of MetS. Other studied socio-economic variables, like education, employment, previous incarceration, and particularly, physical activity, and smoking, showed no differences regarding UA levels.

Using a simple correlation model of continuous variables, we were able to show that the level of UA correlates with the other biochemical parameters studied. Almost all of the correlations were very significantly positive in both the Roma and non-Roma, except ALT in the Roma and Fe in the non-Roma. We found an indirect correlation between the UA levels and HDL cholesterol and ApoA1 in both the Roma and non-Roma participants. On the other hand, a direct correlation exists between the UA and AST, GMT, glu, creatinine, albumin, cysC, TCH, LDL, ApoB1, and ferritin.

Univariate regression showed that UA is a significant predictor of MetS in the whole cohort (unadjusted OR 1.005; 95% CI 1.004–1.007; *p* < 0.0001), also after the adjustment for age, sex, and ethnicity (adjusted OR 1.008; 95% CI 1.005–1.010; *p* < 0.0001). Uric acid remains a significant and independent predictor of MetS in the whole cohort, after excluding the variables defining MetS. The odds of MetS were also increased by age, male sex, elevation of GMT, hsCRP, total cholesterol, and the decrease of ApoA. Poverty was almost a significant predictor of MetS in this model. Interestingly, the Roma ethnicity was not a significant predictor of MetS in this model, along with serum iron and ferritin.

The strengths of our study are the inclusion of a high number of participants randomly selected from the Roma and non-Roma population, inclusion of marginalized Roma people with high barriers to health care, and a thorough examination of the socio-economic background. Its limitations are the cross-sectional design, which limited us from evaluating the relationship between UA and MetS, its components, and the clinical outcomes longitudinally.

Recommendations for practice would be as follows—UA is a routinely available and relatively cheap test that could provide helpful information in the management of patients with metabolic syndrome. However, this test is underused in this indication. Speaking in terms of research—the question remains open if UA is only a marker or a significant causal factor in MetS, therefore further research is needed on the mechanisms connecting the increased levels of UA with MetS and cardiovascular outcomes, and the effects of UA lowering therapy on MetS compensation or cardiovascular outcomes.

## 5. Conclusions

As the socioeconomic determinants of health have become better understood over the past decade, it is becoming clear that societies with greater inequalities are less healthy overall. We were able to show that the prevalence of MetS with all its consequences among the Roma people in Slovakia is higher than in majority population of the country. Moreover, one of the biochemical determinants of MetS, uric acid levels, are significantly lower in the Roma group, but when it comes to cohort with MetS, its levels are also significantly lower compared with the non-Roma group. The levels of UA, besides others, depend on ethnicity, age, and sex, which are independent from each other. Whether UA is a bystander or a central-role player in the pathogenesis of various diseases should be clarified in future studies.

## Figures and Tables

**Table 1:** Studied variables in Roma and non-Roma population.

Studied Variables	Roma	Non-Roma	*p*
	Mean ± Standard Error of Mean or Absolute (Relative) Counts	Mean ± Standard Error of Mean or Absolute (Relative) Counts	
**Demographics**			
Age (in May 2011)	34.67 ± 0.43	33.51 ± 0.37	<0.043
Male sex	159(35.9)	185(45.9)	0.001
**Biochemistry**			
Glucose (mmol/L)	4.84 ± 0.05	4.82 ± 0.030	0.7400
Creatinine (umol)	81.90 ± 0.53	84.95 ± 0.55	<0.0001
Uric acid (umol)	226.54 ± 3.78	259.11 ± 4.22	<0.0001
Albumin (mg/L)	46.60 ± 0.13	47.15 ± 0.15	0.0060
AST (ukat/L)	0.31 ± 0.02	0.33 ± 0.01	<0.0001
ALT (ukat/L)	0.24 ± 0.02	0.25 ± 0.01	0.0040
GMT (ukat/L)	0.43 ± 0.04	0.48 ± 0.03	<0.0001
Cystatin C (mg/L)	0.60 ± 0.01	0.59 ± 0.01	0.3470
Cholesterol (mmol/L)	4.77 ± 0.05	5.13 ± 0.05	<0.0001
Triglycerides (mmol/L)	1.34 ± 0.05	1.24 ± 0.04	0.1090
HDL (mmol/L)	1.08 ± 0.01	1.31 ± 0.02	<0.0001
LDL (mmol/L)	2.51 ± 0.03	2.64 ± 0.03	0.0060
ApoA (mmol/L)	1.52 ± 0.01	1.77 ± 0.02	<0.0001
ApoB100 (mmol/L)	0.77 ± 0.01	0.77 ± 0.01	0.8790
hs-CRP (mg/L)	3.07 ± 0.19	1.98 ± 0.14	<0.0001
Fe (mmol/L)	15.80 ± 0.32	18.56 ± 0.35	<0.0001
Ferritin (mg/L)	209.33 ± 13.59	177.88 ± 10.45	0.0670
**Anthropometrics**			
BMI index	26.57 ± 0.29	24.87 ± 0.22	<0.0001
WHR index	0.87 ± 0.00	0.85 ± 0.02	0.359
Systolic BP (mmHg)	123 ± 1	122 ± 1	0.401
Diastolic BP (mmHg)	75 ± 1	76 ± 0	0.070
**Socioeconomics**			
Employed	46(10.4)	284(73.6)	<0.0001
Poverty	218(48.2)	49(12.2)	<0.0001
Smoking (any)	266(59.8)	110(28.2)	<0.0001
Alcohol (any)	75(17)	64(16.5)	0.844
Education—elementary	360(81.3)	9(2.3)	*p* < 0.0001
Education—apprenticeship	73(16.5)	84(21.4)	
Education—higher	10(2.3)	300(76.3)	
Physical activity (≥2/week)	309(69.9)	222(57.8)	<0.0001
Imprisonment	46(10.3)	4(1.0)	<0.0001

hs-CRP—high sensitivity C-reactive protein; ALT—alanine-aminotransferase; AST—aspartate-aminotransferase; GMT—gamma-glutamyl transferase; HDL—high-density lipoprotein cholesterol; LDL cholesterol—low-density lipoprotein; apoA—apolipoprotein A; apoB100—apolipoprotein B100; Fe—serum iron level; BMI—body mass index; WHR—waist to hip ratio; BP—blood pressure.

**Table 2:** Characteristics of Roma and non-Roma population with and without MetS.

Studied Variables	Roma MetS+	Roma MetS−	Non-Roma MetS+	Non-Roma MetS−	*p*
	Mean ± Standard Error of Mean	Mean ± Standard Error of Mean	Mean ± Standard Error of Mean	Mean ± Standard Error of Mean	
**Demographics**					
Age (in May 2011)	40.21 ± 0.67	32.31 ± 0.49	37.92 ± 0.76	32.37 ± 0.41	<0.0001
Male sex	47(35.9)	111(35.7)	38(47.5)	147(46.1)	0.0200
**Biochemistry**					
Glucose (mmol/L)	5.40 ± 0.14	4.61 ± 0.03	5.16 ± 0.10	4.74 ± 0.03	<0.0001
Creatinine (umol)	83.87 ± 1.07	81.23 ± 0.61	87.72 ± 1.69	84.30 ± 0.54	<0.0001
Uric acid (umol)	251.61 ± 7.87	216.38 ± 4.16	303.64 ± 10.40	247.96 ± 4.40	<0.0001
Albumin (mg/L)	46.22 ± 0.23	46.77 ± 0.16	46.56 ± 0.31	47.31 ± 0.17	0.0020
AST (ukat/L)	0.32 ± 0.03	0.30 ± 0.02	0.36 ± 0.04	0.32 ± 0.01	<0.0001
ALT (ukat/L)	0.26 ± 0.02	0.23 ± 0.02	0.33 ± 0.03	0.23 ± 0.01	<0.0001
GMT (ukat/L)	0.58 ± 0.07	0.37 ± 0.05	0.73 ± 0.10	0.42 ± 0.03	<0.0001
Cystatin C (mg/L)	0.63 ± 0.01	0.59 ± 0.01	0.65 ± 0.02	0.58 ± 0.01	0.0010
Cholesterol (mmol/L)	5.14 ± 0.09	4.63 ± 0.05	5.60 ± 0.11	5.01 ± 0.05	<0.0001
Triglycerides (mmol/L)	2.14 ± 0.12	1.01 ± 0.03	2.09 ± 0.13	1.03 ± 0.03	<0.0001
HDL (mmol/L)	0.90 ± 0.02	1.16 ± 0.02	1.06 ± 0.02	1.38 ± 0.02	<0.0001
LDL (mmol/L)	2.73 ± 0.06	2.42 ± 0.04	3.01 ± 0.08	2.55 ± 0.04	<0.0001
ApoA (mmol/L)	1.42 ± 0.02	1.56 ± 0.02	1.63 ± 0.03	1.81 ± 0.02	<0.0001
ApoB100 (mmol/L)	0.87 ± 0.02	0.72 ± 0.01	0.94 ± 0.03	0.73 ± 0.01	<0.0001
hsCRP (mg/L)	5.20 ± 0.44	2.20 ± 0.18	3.40 ± 0.46	1.62 ± 0.13	<0.0001
Fe (mmol/L)	14.23 ± 0.52	16.51 ± 0.39	18.17 ± 0.63	18.62 ± 0.40	<0.0001
Ferritin (mg/L)	266.35 ± 25.20	187.70 ± 16.31	255.65 ± 32.68	158.84 ± 10.00	<0.0001
**Anthropometrics**					
BMI index	31.61 ± 0.50	24.42 ± 0.27	29.58 ± 0.42	23.63 ± 0.21	<0.0001
WHR index	0.92 ± 0.01	0.85 ± 0.00	0.90 ± 0.01	0.84 ± 0.03	0.0580
Systolic BP (mmHg)	136 ± 2	117 ± 1	132 ± 2	119 ± 1	<0.0001
Diastolic BP (mmHg)	84 ± 1	71 ± 1	83 ± 1	74 ± 1	<0.0001
**Socioeconomics**					
Employed	16(12.7)	29(9.4)	57(73.1)	227(73.7)	<0.0001
Poverty	73(55.7)	139(44.7)	16(20)	32(10)	<0.0001
Smoking (any)	74(57.8)	184(59.7)	25(31.6)	85(27.4)	<0.0001
Alcohol (any)	19(15)	55(18)	15(19.5)	48(15.5)	0.7010
Education—elementary	103(81.1)	250(81.4)	3(3.8)	6(1.9)	<0.0001
Education—apprenticeship	21(16.5)	51(16.6)	24(30)	60(19.2)	
Education—higher	3(2.4)	6(2.0)	53(66.3)	246(78.8)	
Physical activity (≥2/week)	93(73.2)	211(69)	45(57.7)	177(58.0)	0.0030
Imprisonment	12(9.4)	33(10.7)	2(2.5)	2(0.6)	<0.0001

hs-CRP—high sensitivity C-reactive protein; ALT—alanine-aminotransferase; AST—aspartate-aminotransferase; GMT—gamma-glutamyl transferase; HDL—high-density lipoprotein cholesterol; LDL cholesterol—low-density lipoprotein; apoA—apolipoprotein A; apoB100—apolipoprotein B100; Fe—serum iron level; BMI—body mass index; WHR—waist to hip ratio; BP—blood pressure.

**Table 3:** Uric acid (UA) levels categorized by different socio-economic variables for the Roma and non-Roma participants.

Socio-Economic Variables		Roma	*p*	Non-Roma	*p*
		Mean ± SEM		Mean ± SEM	
**Poverty**	No	219 ± 5.2	0.065	260 ± 4.5	0.564
	Yes	233 ± 5.5		252 ± 12.8	
**Alcohol intake**	Less than once a month, or never	222 ± 4.2	0.052	254 ± 4.5	0.007
	Once in a month, week, or daily	241 ± 9.1		291 ± 12.7	
**Education**	Elementary	223 ± 4.1	0.204	280.5 ± 13.4	0.691
	Apprenticeship	239 ± 10.4		263 ± 9.9	
	Higher	247 ± 25.5		258 ± 4.9	
**Smoking**	No	233 ± 6.3	0.105	258 ± 5	0.428
	Yes	220 ± 4.7		263 ± 8.4	
**Physical activity**	Once a week or less	225 ± 4.4	0.983	261 ± 5.6	0.858
	2–3 times a week or more	225 ± 7.1		260 ± 6.9	
**Employed**	No	226 ± 3.9	0.489	255 ± 9.0	0.464
	Yes	217 ± 12.5		262 ± 4.9	
**Imprisoned previously**	No	221 ± 3.9	0.013	260 ± 28.7	0.974
	Yes	259±14		260 ± 4.3	

**Table 4:** Pearson correlation of UA and other biochemical parameters.

Biochemical Parameters	Roma		Non-Roma	
	Corr Coeff	*p*	Corr Coeff	*p*
Glucose (mmol/L)	0.159	<0.0001	0.169	<0.0001
Creatinine (umol)	0.391	<0.0001	0.532	<0.0001
Albumin (mg/L)	0.203	<0.0001	0.128	0.0110
AST (ukat/L)	0.118	0.0130	0.245	<0.0001
ALT (ukat/L)	0.087	0.066	0.312	<0.0001
GMT (ukat/L)	0.122	0.010	0.307	<0.0001
Cystatin C (mg/L)	0.162	<0.0001	0.226	<0.0001
Cholesterol (mmol/L)	0.111	0.019	0.183	<0.0001
Triglycerides (mmol/L)	0.317	<0.0001	0.284	<0.0001
HDL (mmol/L)	−0.211	<0.0001	−0.222	<0.0001
LDL (mmol/L)	0.104	0.0290	0.220	<0.0001
ApoA (mmol/L)	−0.179	<0.0001	−0.223	<0.0001
ApoB100 (mmol/L)	0.125	0.0080	0.208	<0.0001
hsCRP (mg/L)	0.259	<0.0001	0.167	<0.0001
Fe (mmol/L)	0.123	0.0090	0.086	0.0860
Ferritin (mg/L)	0.349	<0.0001	0.422	<0.0001

hs-CRP—high sensitivity C-reactive protein; ALT—alanine-aminotransferase; AST—aspartate-aminotransferase; GMT—gamma-glutamyl transferase; HDL—high-density lipoprotein cholesterol; LDL cholesterol—low-density lipoprotein; apoA—apolipoprotein A; apoB100—apolipoprotein B100; Fe—serum iron level; BMI—body mass index; WHR—waist to hip ratio; BP—blood pressure.

**Table 5:** Uric acid levels in subgroups according to fulfilled metabolic syndrome criteria.

MetS Criteria		Roma		nonRoma	
		Mean ± SEM	*p*	Mean ± SEM	*p*
**Glucose criterium**	No	223 ± 3.9		256 ± 4.3	
	Yes	261 ± 12.0	0.0020	298 ± 16.5	0.0080
**Low HDL criterium**	No	221 ± 7.0		254 ± 5.0	
	Yes	228 ± 4.5	0.3300	269 ± 7.7	0.0730
**Obesity criterium**	No	210 ± 5.4		243 ± 77.7	
	Yes	239 ± 5.2	<0.0001	280 ± 6.7	<0.0001
**TG criterium**	No	216 ± 3.9		247 ± 4.3	
	Yes	260 ± 9.2	<0.0001	308.8 ± 10.8	<0.0001
**BP criterium**	No	217 ± 4.5		245 ± 4.9	
	Yes	251 ± 7.1	<0.0001	290 ± 7.7	<0.0001

Obesity criterium—waist circumference ≥94 cm for males and ≥80 cm for females or BMI >30 kg/m^2^; low HDL criterium—HDL level <1.03 mmol/L (40 mg/dL) in males and <1.29 mmol/L (50 mg/dL) in females, or specific treatment for these lipid abnormalities; TG criterium—TG level ≥1.7 mmol/L (150 mg/dL) or specific treatment for this lipid abnormality; glucose criterium—glycemia ≥5.6 mmol/L (100 mg/dL) or previously diagnosed type 2 diabetes; BP criterium—systolic BP ≥130 mmHg or diastolic BP ≥85 mmHg or treatment of previously diagnosed hypertension. MetS—metabolic syndrome.

**Table 6:** Fully adjusted multivariate regression model with Metabolic syndrome as a dependent variable.

Variables with Significant Difference between MetS and without MetS	Sig.	Exp(B)	95% CI for EXP(B)	
			Lower	Upper
Uric Acid (umol)	0.005	1.004	1.001	1.007
Age (years)	<0.0001	1.095	1.067	1.124
Female sex	0.001	0.406	0.242	0.682
GMT (ukat/L)	0.007	1.450	1.108	1.897
hsCRP (mg/L)	<0.0001	1.151	1.086	1.219
Fe (mmol/L)	0.419	0.987	0.956	1.019
Ferritin (mg/L)	0.327	1.000	1.000	1.001
Roma ethnicity	0.261	0.758	0.468	1.228
Cholesterol (mmol/L)	<0.0001	1.759	1.410	2.194
Apolipoprotein A	<0.0001	0.048	0.021	0.107
Poverty	0.053	1.535	0.995	2.367
Constant	<0.0001	0.031		

hs-CRP—high sensitivity C-reactive protein; GMT—gamma-glutamyl transferase; Fe—serum iron level. MetS—metabolic syndrome.
